# Who wants to be a psychiatrist? Northern Ireland foundation doctors (2006–2018) are positive toward psychiatry as career choice

**DOI:** 10.1192/bjb.2019.86

**Published:** 2020-08

**Authors:** Michael Doris, Amy Grimason, Damien Hughes, Edel O'Neill, Lorraine Parks, Angela Carragher

**Affiliations:** 1 Northern Ireland Foundation School, Northern Ireland Medical and Dental Training Agency, UK; 2 Psychiatry, Belfast Health and Social Care Trust, UK; 3 Psychiatry, Northern Ireland Medical and Dental Training Agency, UK; 4 Psychiatry, Southern Health and Social Care Trust, UK; 5 Anaesthetics, Southern Health and Social Care Trust, UK

**Keywords:** Qualitative research, education and training, recruitment

## Abstract

**Aims and method:**

Northern Ireland presents itself as an anomaly – a region in which only 31.8% of doctors enter into any training programme after completion of the Foundation Programme, but where Core Psychiatry has been consistently oversubscribed. Here, we aim to find what other regions can learn from this success. All doctors of any grade, working in psychiatry, who had been though the Foundation Programme were questioned on their motivations for becoming a psychiatry trainee.

**Results:**

Sixty-two doctors currently working in psychiatry responded, including over 60% of current trainees, and 45% stated they had not considered a career in psychiatry before their foundation attachment. Over 80% preferred foundation placements in FY2 only, rather than in either foundation year 1 or FY2.

**Clinical implications:**

This survey identifies that for the majority of people who ultimately chose to train in psychiatry, in a region that has consistently attracted candidates to core and higher level training, completion of a foundation psychiatry post was an influencing factor in this decision. A strong majority of doctors prefer the foundation psychiatry placement to be offered in FY2.

Recruitment into core psychiatry training in the UK has been a longstanding area of concern.^1^ Despite the success of the Royal College of Psychiatrist's ‘Choose Psychiatry’ scheme and the subsequent increase in the fill rate in 2018,^2^ there still remains 18% of posts that are not filled nationally. There are, however, variations based on geographical area, with Northern Ireland consistently achieving a 90–100% fill rate at core recruitment.

Following the Collins Review^3^ in 2010 highlighting the need for more community foundation placements and the Royal College of Psychiatrists recruitment strategy^4^ of 2011, one option put forward was to increase exposure to psychiatry during the Foundation Programme. A target was set to have 22.5% of all foundation year 1 (FY1) and 22.5% of all foundation year 2 (FY2) doctors offered placements in psychiatry. Northern Ireland is the only region of the UK that does not offer any placements in FY1, but it does offer a 4-month psychiatry placement to approximately 33% of FY2 doctors.

In this survey we aimed to explore why psychiatry doctors working in Northern Ireland chose their specialty and whether having a psychiatry foundation post in FY2 influenced their career choice. We also examined the opinions of the doctors who chose psychiatry following an FY2 placement on when psychiatry should be offered in the Foundation Programme.

## Method

### Participants

The participants for this survey were doctors working in psychiatry in Northern Ireland who had completed Foundation Programme training since its establishment in 2006. This consisted of a group of psychiatry core trainees, speciality trainees (in all areas of psychiatry) and consultants.

### Survey design

The survey was designed using the SurveyMonkey online questionnaire system, following collaboration between the Northern Ireland Foundation School and the Northern Ireland School of Psychiatry.

**Fig. 1:**
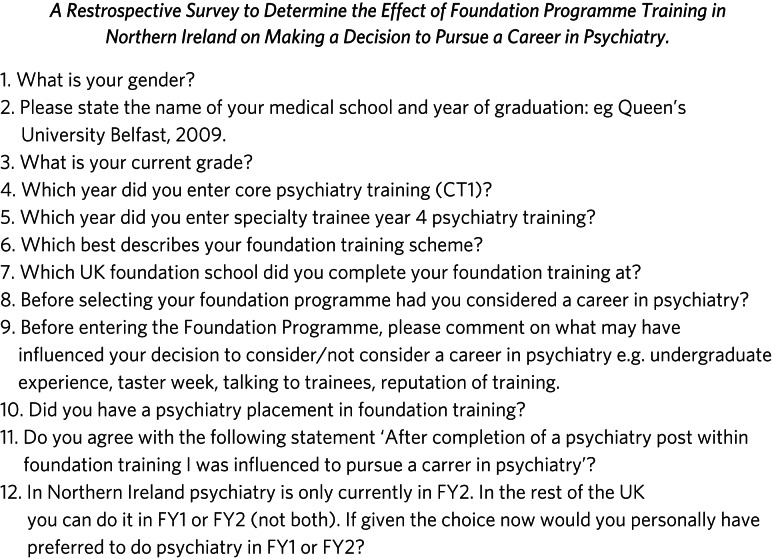
Survey questionnaire. FY1, foundation year 1; FY2, foundation year 2.

The survey questionnaire (Fig. 1) consisted of 12 questions establishing baseline demographic data with regards to gender, medical school, year of graduation, current grade, year entering into core psychiatry training and, if applicable, year entering specialist training in psychiatry. There were questions that established the trajectory of foundation training and if this was planned, coordinated by the UK Foundation Programme and the duration of their foundation programme. Respondents were asked if they had considered a career in psychiatry before selecting their foundation programme and what may have influenced their decision in this (i.e. undergraduate experience, training reputation). The respondents were then asked if they had a psychiatry placement in foundation training and if they agreed with the statement, ‘After completion of a psychiatry post within foundation training I was influenced to pursue a career in psychiatry’. The final question was examining the preferences of respondents in the option of having psychiatry available as a preference in FY1 (which is not currently offered in Northern Ireland but is offered in other parts of the UK). The nature of the survey resulted in retrospective data being collected.

### Distribution

It was an open and voluntary survey. The survey was populated via an email from the current Head of School of Psychiatry to all doctors who had been enlisted in psychiatry training from 2006 (i.e. after the first full 2-year cycle of the Foundation Programme). The survey was open for 3 weeks. This email list was obtained from records within the School of Psychiatry at the local deanery (Northern Ireland Medical and Dental Training Agency; NIMDTA). Reminders were sent via email and social media to encourage completion. The survey was sent to 92 psychiatry trainees and 25 specialty doctors/consultants; as updated lists of consultant emails are not held by the deanery, emails may not have reached all the doctors who were no longer in training, so the figure of 25 is approximate.

### Analysis

Following the closure of the online survey, the results were collated in their entirety for evaluation by the authors. Quantitative data was exported to Microsoft Excel 2016 (v.16.0) for analysis. As a portion of the data was from ‘white space’ or free-text questions and thus qualitative in nature, these were assessed for common themes.

## Results

A total of 67 participants responded to the survey, and demographic data is represented in Table 1. Of the 12 questions, 7 were answered by all respondents (questions 2, 3, 4, 6, 7, 8 and 10). Of the remaining questions, there were 66 responses, with the exception of one in which there were 65 responses (question 11). The majority of the respondents were female (*n* = 42, 63.6%).

**Table 1:** Demographic data

	Total (*n* = 67)
Gender	
Female	42 (63.6%)
Male	24 (34.3%)
Current grade	
Specialty trainee	32 (47.8%)
Core trainee	26 (38.8%)
Non-trainee doctors (i.e. consultant, specialty doctor)	9 (13.4%)
Foundation scheme	
2-year planned programme	60 (89.5%)
Recruited to a 1-year standalone FY2 programme	5 (7.5%)
Other	2 (3%)
Psychiatry placement in Foundation Programme	
In FY1	3 (4.5%)
In FY2 (first placement)	33 (49.3%)
In FY2 (second placement)	13 (19.4%)
In FY2 (third placement)	11 (16.4%)
No psychiatry placement	10 (10.4%)
Considering psychiatry before Foundation Programme	
Yes	37 (55.2%)
No	30 (44.8%)

FY2, foundation year 2; FY1, foundation year 1.

A high proportion of respondents had completed their undergraduate training at Queen's University Belfast (*n* = 62, 92.5%) and year of graduation ranged from 2006 to 2016. The vast majority of respondents had completed their foundation training within the deanery in Northern Ireland, NIMDTA (*n* = 63, 94.0%).

**Fig. 2:**
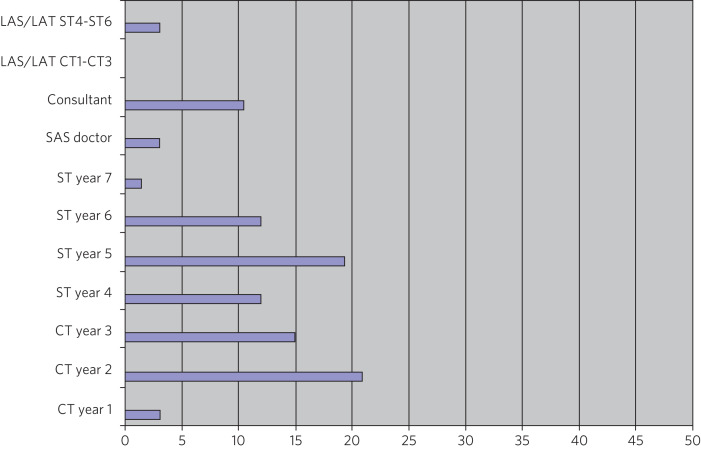
Grade breakdown of participants, *n* = 62. LAS, Locum Approved Service; LAT, Locum Approved Training; SAS, Specialty and Associate Specialist; ST, Senior Trainee; CT, Core Trainee.

The majority of respondents were in training; almost half of respondents were currently in specialty training (*n* = 32, 47.8%), with a slightly smaller percentage in core training (*n* = 26, 38.8%). Consultants made up a small proportion (*n* = 7, 10.4%), with a smaller number of specialty doctors and associate specialists (*n* = 2, 3%). Figure 2 shows the distribution across training levels.

The year of entering core psychiatry training ranged from 2007 to 2017, and year of entering into specialty training ranged from 2011 to 2018 for those that this was applicable to.

The vast majority of respondents were recruited through the Foundation Programme UK system on a planned 2-year Foundation Training Scheme (*n* = 60, 89.6%). A small number (*n* = 5, 7.5%) were recruited through the Foundation Programme UK system on a single 1-year FY2 programme.

The majority of participants had a foundation placement in psychiatry (*n* = 60, 89.5%). The breakdown of when participants had their placements is shown in Table 1. Those who had a psychiatry placement in FY1 had completed their foundation training outside of Northern Ireland.

When asked if they had considered a career in psychiatry before selecting their foundation programme, 55.2% of respondents (*n* = 37) reported they had and 44.8% (*n* = 30) reported they had not. When asked what influenced their decision to choose (or not choose) to pursue a career in psychiatry before commencing foundation training, the most commonly cited theme was undergraduate experience of psychiatry (*n* = 47, 70.1%). Generally, the majority of respondents who cited this as a factor reported a positive contributor (*n* = 34, 72.3%) toward their choice to pursue psychiatry as a career. The second most commonly cited factor was the positive reputation of psychiatry training in Northern Ireland (*n* = 20, 29.9%). This factor encompassed the positive reputation of training and also of having positive interactions with trainees and consultants in their undergraduate training. The third most commonly cited factor positive influencing people's decision to enter psychiatry was of having an interest in the subject (*n* = 11, 16.4%).

Other less commonly cited positive factors were of having a qualification in a related field (such as psychology), enjoyment of a psychiatry locum post, the expectation of a positive work–life balance over the course of their career, media and mental health group influences and future job prospects.

The most commonly cited reasons that led people originally to not consider psychiatry as a career was a negative or ambivalent undergraduate experience (*n* = 13, 19.4%) Those that were negative or ambivalent reported not enjoying their experience, feeling daunted by how different psychiatry was from other medical specialties and also by their experiences in in-patient settings in undergraduate training.

Exposure to negative opinions of psychiatry in placements in other specialties was also cited as a reason people did not consider psychiatry. In response to the statement ‘After the completion of a psychiatry post within foundation training I was influenced to pursue a career in psychiatry’, the majority of respondents agreed with it (*n* = 60, 92.%).

The final questions gathered the views of respondents as to whether psychiatry should be offered to FY1 doctors. The majority of respondents (*n* = 54, 81.8%) felt that psychiatry should only be offered as an FY2 rotation in the Foundation Programme. By far the most commonly cited reason for this was to allow for the development of general skills and improve general medical and surgical knowledge in FY1 (*n* = 42, 77.8%). Many felt this to be important as often psychiatry units were isolated from acute hospitals and out-of-hours work in psychiatry is often non-resident with senior assistance also being non-resident. One respondent felt that a job in psychiatry for an FY1 may not be representative of a psychiatry experience as the doctor would likely complete more administrative and medical tasks. Another respondent felt that FY2 was when doctors are more likely to be considering their career options.

For those who reported wishing to have a psychiatry placement in FY1 (*n* = 12, 18.2%) the most common reason for this was that it would give doctors earlier exposure, which may encourage them to apply for psychiatry (*n* = 8, 80.0%). Some reported they had missed an application process owing to having psychiatry as their last rotation in their FY2 year. Two respondents reported that psychiatry would be useful in FY1 to give doctors early exposure to a more holistic care approach.

## Discussion

This survey adds to previous findings^5–7^ that exposure to psychiatry in foundation training is a powerful tool in recruiting doctors to work in the field. Remarkably, of doctors working in psychiatry in Northern Ireland, 45% had not considered a career in psychiatry before their foundation placement, and 92.31% went on to agree with the statement ‘After the completion of a psychiatry post within foundation training I was influenced to pursue a career in psychiatry’.

Doctors working in psychiatry spoke positively of the undergraduate experience and the close local ties between the medical school and the deanery in establishing a programme that encouraged them to apply. Northern Ireland has one medical school, Queen's University Belfast, of which around 70–80% of foundation doctors working in Northern Ireland graduate from.

In considering recruitment strategies, the proportion of those who are attracted after undergraduate level should not be underestimated: in a survey including 51 core trainees, Denman *et al*^8^ found that most chose psychiatry during their foundation placement as opposed to as an undergraduate, a finding that resonates with our conclusion of the importance and influence of foundation training experience to the specialty's recruitment. There has been extensive research into the factors that affect recruitment into psychiatry and our findings are similar to those identified in previous studies.^9^

The importance of a positive experience of psychiatry in medical school has been highlighted in numerous studies. Mukherjee *et al*^10^ identified the significance of a genuine interest in the subject as being important to recruitment and proposed that the way some undergraduate placements are structured (mainly in acute in-patient settings) expose students to complex patients who are potentially the most unwell, perhaps resulting in a skewed perception of those with mental illness. They highlight the need for more doctors to complete foundation placements in psychiatry and that the process of choosing a specialty within a year of qualifying disadvantaged psychiatry in terms of recruitment, it being a career choice for ‘late bloomers'. This was specifically highlighted in our study with one participant missing the application window owing to not having a psychiatry placement until the end of their foundation programme, forcing them to wait a further year before applying. They also identified a perception that psychiatry was less scientific and of lower status than other areas of medicine as an important factor with regards to negatively affecting recruitment.

Given the unique cohort of doctors in Northern Ireland, we looked to establish what attitudes were toward the current approach of having 100% of psychiatry placements in FY2. An overwhelming 81.82% felt that this was what they would personally choose. They speculated that a FY1 doctor may be seen as the ‘medical doctor’ and may not get the same breadth of experience of acute psychiatric care as an FY2 doctor.

This survey identifies that a strong majority of doctors prefer the foundation psychiatry placement to be offered in FY2, from a region that has consistently attracted candidates to core and higher level training. As a region without an FY1 placement, the generalisability of the results must be considered as a weakness of the survey. It could be argued that those who stated that they would prefer psychiatry in FY2 have limited frame of reference for such an argument.

We believe there are a number of reasons that could help explain why recruitment in Northern Ireland is higher than in other regions in the UK. As we have already alluded to, there is a sense of collegiality in Northern Ireland with regards to the speciality. Northern Ireland has one medical school, one medical training body and one Royal College of Psychiatrists headquarters, allowing for the promotion of psychiatry to be streamlined and coordinated between these entities.

There has been a concerted effort to promote psychiatry in undergraduate training and to ensure that this is of good quality. Medical students have a 6-week placement in psychiatry in their 4th year and also opportunities earlier in their studies to partake in psychiatry-themed, student-selected modules. Enthusiastic psychiatry trainees are also visible within the undergraduate teaching, delivering teaching sessions to a variety of undergraduate years, promoting the speciality from the start of the curriculum. Queen's University produces proportionally more psychiatry doctors than any other university in the UK, which is indicative of the successes of these efforts.

Following a positive undergraduate experience, more doctors then experience a FY2 placement in psychiatry than in other regions in the UK, which this survey has shown to be an important influencing factor for those not previously considering the specialty. The structuring and delivery of psychiatry training is potentially another important reason why Northern Ireland has higher recruitment figures: psychiatry training has a good reputation in Northern Ireland because of its weekly protected teaching time, with subsequent examination pass rates that are above the national average.

Nationally there is evidence of green shoots in psychiatry recruitment^9^ and there is a momentum being built by the Choose Psychiatry movement. However, there is still cause for concern with recruitment of doctors into psychiatry and continued efforts are needed to build upon the progress already made. Here we show that a strong foundation programme in Northern Ireland, focused on FY2, showcases the specialty as a beacon for recruitment in the UK. This is reinforced by strong links at local university and college level, supported by trainers who buy into personal and professional development and trainees who provide role modelling at an early stage for trainee doctors.

## About the authors

**Michael Doris** is a higher trainee in learning disability psychiatry with Belfast Health and Social Care Trust, and an ADEPT Leadership Fellow at Northern Ireland Medical and Dental Training Agency, Northern Ireland. **Amy Grimason** is a higher trainee in forensic psychiatry with Belfast Health and Social Care Trust, Northern Ireland. **Damien Hughes** is a consultant psychiatrist working in learning disability psychiatry with Belfast Health and Social Care Trust, and Head of the School for Psychiatry at Northern Ireland Medical and Dental Training Agency, Northern Ireland. **Edel O'Neill** is a consultant psychiatrist working in learning disability psychiatry with Southern Health and Social Care Trust, Northern Ireland. **Lorraine Parks** is a consultant anaesthetist with Southern Health and Social Care Trust, and Associate Postgraduate Dean for the Northern Ireland Foundation School at Northern Ireland Medical and Dental Training Agency, Northern Ireland. **Angela Carragher** is a consultant surgeon and former Associate Postgraduate Dean for the Northern Ireland Foundation School at Northern Ireland Medical and Dental Training Agency, Northern Ireland.
